# Not Just 9-1-1: The Expanded Work Environment of United States Emergency Medical Service Clinicians

**DOI:** 10.7759/cureus.72779

**Published:** 2024-10-31

**Authors:** Christopher B Gage, Jonathan R Powell, Christine B Cooke, Jacob C Kamholz, Shea van den Bergh, Jordan D Kurth, Ashish Panchal

**Affiliations:** 1 Emergency Medicine, National Registry of Emergency Medical Technicians, Columbus, USA; 2 General Internal Medicine, Penn State College of Medicine, Hershey, USA; 3 Emergency Medicine, The Ohio State University Wexner Medical Center, Columbus, USA

**Keywords:** advanced emergency medical technicians, emergency medical technicians, ems workforce, paramedics, primary job roles

## Abstract

Introduction: Recently, there have been annual increases in emergency medical service (EMS) demand with concurrent clinician shortages. Understanding the diverse number of EMS clinicians available for emergent roles is vital for planning and resource management. Our study aims to understand the roles and environments of US EMS clinicians.

Methods: This cross-sectional study analyzed nationally certified civilian US EMS clinicians recertifying from October 2021 to April 2022, ages 18-85, with at least one EMS job. Respondents answered questions regarding their primary and secondary EMS roles, including emergent response (with/without 9-1-1), medical transport (non-emergent), clinical services, mobile integrated health (MIH), or none of the above. Next, respondents were asked about the number of jobs needed to make ends meet. All responses were combined with self-reported National Registry profile data (e.g., age, sex). Descriptive statistics were performed.

Results: The study included 33,335 EMS clinicians (response rate: 34.0%). The primary role reported was emergent response with 25,086 (75.3%), including ground ambulance and non-ambulance response. Clinical service roles were reported by 2,427 (7.3%), including settings such as the emergency department and outpatient clinics. Medical transport roles accounted for 2,346 (7.0%), including ground interfacility and critical ground care. Educators and administrators made up 1,453 (4.4%). Overall, roles varied by sex, and 48.8% of respondents reported needing more than one job to make ends meet.

Conclusions: Our evaluation highlights various US EMS clinician roles. These findings suggest the need for continued focus and attention on EMS roles, compensation structures, and sex distributions to ensure a resilient, diverse, and adequately supported EMS workforce.

## Introduction

Emergency medical service (EMS) systems are a critical pathway of access to acute prehospital care in the US [1], with over 54 million documented activations in 2023 [2]. The demand for EMS is increasing annually, with workforce projections showing 5% growth from 2022 to 2030 [3]. However, clinician shortages in many US communities contribute to challenges in delivering timely and effective care [4-7]. Expanded roles of EMS in the healthcare continuum include integrating EMS clinicians into various healthcare specialties and settings outside the prehospital field [8]. Though these expanded work environments are an important aspect of the long-term vision for EMS clinicians, this gradual expansion of EMS clinician roles beyond 9-1-1 emergent care may have unintended consequences that influence workforce dynamics and the effectiveness of EMS agencies in meeting community and patient needs [9].

One challenge in understanding the extent of this issue is the lack of clear definitions for these evolving US EMS clinician work environments [10,11], including the setting, social, and physical conditions where an individual works. In EMS, work environments are closely linked to the role fulfilled [12]. Prehospital 9-1-1 emergency response roles place the clinician in variable and unstable spaces. In contrast, roles in hospital care provide a stable, controlled, and reproducible clinical space. These roles have shifted and expanded in EMS, and preliminary evaluations have noted an increasing percentage of these clinicians being employed in hospital settings [13,14]. Additional role expansions, including delivering care in home health settings, physician outpatient practices, and industry-specific areas (e.g., offshore oil rigs, event settings, security detachments, and construction), further diversify and potentially complicate our understanding of the true EMS care delivery landscape [14]. Unfortunately, there is a lack of data on the specific roles where EMS clinicians deliver care. This lack of data limits our understanding of how effectively EMS systems can respond to emergencies, as well as our ability to effectively forecast the growth potential and demand for the future EMS workforce.

In the context of evolving workforce dynamics and increasing population needs, a deeper understanding of the work environment roles where EMS clinicians practice is essential. In this study, we address this issue by evaluating the work environment roles fulfilled by EMS clinicians in the US. We leverage the most comprehensive and available US EMS database to survey and understand the primary and secondary environment roles of EMS clinicians.

## Materials and methods

Study design, setting, and participants

This study is a cross-sectional survey aimed to assess the work environments and roles of nationally certified EMS clinicians in the US. The study evaluates work environments and roles in the context of participant demographics and EMS workforce characteristics. Surveys were administered as an optional part of the National Registry of Emergency Medical Technicians (National Registry) recertification process. This study was deemed exempt by the American Institutes of Research Institutional Review Board (project number: EX00572).

The National Registry is a nonprofit organization that certifies EMS clinicians in more than 46 states, territories, and federal agencies at one or more certification levels [15]. We invited all EMS clinicians who completed a National Registry recertification application between October 2021 and April 2022 to participate in a voluntary survey after submitting their biennial recertification application. The survey was administered using the Alchemer Surveys platform, with approximately 100,000 EMS clinicians eligible to participate. In previous peer-reviewed recertification dataset evaluations, each recertifying cohort has been demonstrated to be a representative sample of the National EMS Certification database [15].

Nationally registered EMS clinician levels include emergency medical responder (EMR), emergency medical technician (EMT), advanced emergency medical technician (AEMT), and paramedic [15]. This study includes only civilian EMTs, AEMTs, and paramedics, ages 18-85, with at least one EMS job. This population was selected to represent the civilian prehospital workforce in the US that responds to and transports patients to definitive care. Thus, we excluded those EMS clinicians reporting primary military affiliations or EMR certification levels.

Measures

Demographic characteristics were measured similarly to previous evaluations [16] and included sex (designated as male or female), minority status, and education level. Minority status included participants who self-identified as Black or African American, Asian, Hispanic, Latino, Native Hawaiian, or Pacific Islander. Due to the small proportion of minority EMS clinicians, minority status was dichotomized into non-minority (White, non-Hispanic) or minority. Education level was categorized as high school or general educational development (GED) or less, some college experience, associate’s degree, or bachelor’s degree or more.

Job characteristics were evaluated by certification level (EMT, AEMT, or paramedic). Years of experience were measured as a continuous variable. Primary EMS employment status was full-time or part-time, and whether that job was mainly volunteer (yes/no). The number of EMS jobs was self-reported as the number of EMS jobs the respondent currently holds and categorized as 1 or 2+. Self-reported community size was defined as urban/suburban and rural as defined by the US Census classification, with urban areas having populations of 50,000 or more, urban clusters (suburban) with populations of 2,500 to 50,000, and rural areas as those areas not included as urban or suburban (<2,500).

Outcomes

Our primary outcome was evaluating EMS clinicians’ primary work environment roles, regardless of patient care duties. The primary EMS role was self-selected by each respondent and broadly categorized as emergent response (with/without 9-1-1), medical transport (non-emergent), clinical services, mobile integrated health (MIH), and none of the above. Next, respondents further defined their roles by selecting sub-categories for each primary role. Finally, they answered questions about the number of EMS jobs they held, how many jobs (EMS or not) they needed to make ends meet, and whether they had secondary roles within their main EMS agency.

Data analyses

We calculated descriptive statistics to describe population characteristics. Age and experience were analyzed as continuous variables, and all other variables were treated categorically. Continuous variables were described with median and interquartile ranges (median (IQR)). Categorial variables were expressed as a proportion (%) of each group’s total.

Additionally, recognizing that disparities by sex exist among EMS clinicians, we also conducted a stratified analysis by biological sex for primary roles [17]. All analyses were completed using Stata/SE version 17 (StataCorp LP, College Station, TX). To manage potential sampling bias and to ensure our estimates better reflect the characteristics of the overall population, we conducted survey weighting based on the nationally certified EMS population demographics identified by the National Registry’s recertification dataset. Survey weights were calculated for demographic variables: age, sex, race, education, and agency type. Weights were computed as the ratio of national to survey population proportions of each variable’s subgroups (e.g., education: high school/GED, some college, associates, etc.). Composite base weights were then assigned to every individual to account for the multiple variables/subgroups in the dataset.

## Results

**Figure 1:**
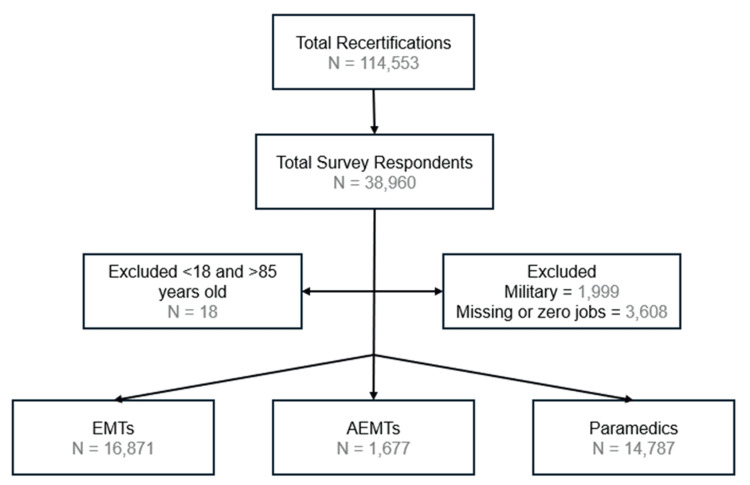
Flow chart with populations AEMT: advanced emergency medical technicians; EMT: emergency medical technicians

Between October 2021 and April 2022, 114,553 EMS clinicians recertified their National Registry certifications. After excluding ages <18 and >85, those with primary military roles, and zero or missing jobs, 33,335 were analyzed (Figure 1). A total of 16,871 EMTs, 1,677 AEMTs, and 14,787 paramedics were included in the analysis.

Demographics of the population are noted in Table 1. Males were most common among all certification levels but were more prominent as the certification level scope of practice increased from EMT to paramedic. Median age ranged from 33 to 39 and increased with increasing certification levels. EMTs and AEMTs had higher respondent percentages with some college and bachelor’s degrees compared to paramedics with a higher percentage of bachelor’s degrees. Most respondents worked full-time with their primary agencies, which increased with certification levels, compared to those volunteering, which decreased with certification levels (Table 1). EMTs worked at fewer EMS agencies compared to AEMTs and paramedics. Respondents across all levels mainly worked in urban settings.

**Table 1:** Demographic and job characteristics of respondents AEMT: advanced emergency medical technicians; EMT: emergency medical technicians; HS/GED: high school/general educational development; IQR: interquartile range

Characteristics	EMT N = 16,871 N (%)	AEMT N = 1,677 N (%)	Paramedic N = 14,787 N (%)
Sex			
Male	11,650 (69.1)	1,179 (70.3)	11,669 (78.9)
Female	5,149 (30.5)	489 (29.2)	3,090 (20.9)
Missing	72	9	28
Age (Median, IQR)	33 (26-43)	35 (28-44)	39 (32-48)
Minority Status			
Non-Hispanic White	13,300 (78.8)	1,430 (85.3)	12,792 (86.5)
Minority	3,287 (19.5)	214 (12.8)	1,761 (11.9)
Missing	284	33	234
Education			
HS/GED or less	3,069 (18.2)	285 (17.0)	1,452 (9.8)
Some College	5,757 (34.1)	633 (37.7)	4,379 (29.6)
Associate degree	2,524 (15.0)	291 (17.4)	4,239 (28.7)
Bachelor’s degree or more	5,493 (32.6)	468 (27.9)	4,714 (31.9)
Missing	28	0	3
Median Years Experience (IQR)	3 (2-5)	4 (2-5)	4 (3-6)
Primary EMS Employment Status			
Full-time-yes	10,905 (64.6)	1,209 (72.1)	12,822 (86.7)
Volunteer-yes	3,421 (20.3)	223 (13.3)	473 (3.2)
Number of EMS Jobs			
1	12,992 (77.0)	1,128 (67.3)	10,103 (68.3)
2+	3,879 (23.0)	549 (32.7)	4,684 (31.7)
Community size			
Urban/Suburban	13,883 (82.3)	1,398 (83.4)	13,503 (91.3)
Rural	2,415 (14.3)	225 (13.4)	860 (5.8)
Missing	573	54	424
Jobs to make ends meet			
1	8,104 (48.0)	685 (40.8)	7,238 (48.9)
2+	8,005 (47.4)	964 (57.5)	7,285 (49.3)
Missing	762	28	264

Regardless of certification level, most respondents primarily performed emergent response roles (Table 2). For the second most common primary roles, EMTs worked in clinical services, with 1,466 representing 8.7%, while AEMTs and paramedics were also in clinical services, with 96 and 864 representing 5.7% and 5.8%, respectively. The same top two sub-category roles were consistent across certification levels. When totaled, ground ambulance was the largest overall sub-category role for all certifications: 39.3% for EMTs, 58.7% for AEMTs, and 58.5% for paramedics (Table 3). Additionally, totaled sub-categories other than ground ambulance and non-ambulance response (rescue squad, fire apparatus) accounted for 24.6% of EMTs, 20.1% of AEMTs, and 12.8% of paramedics’ primary roles. Almost half of all EMTs (47.4%) and paramedics (49.3%) and over half of AEMTs (57.5%) reported needing more than one job to make ends meet (Table 1).

**Table 2:** EMS clinician work roles performed over the last 12 months AEMT: advanced emergency medical technician; EMT: emergency medical technician

Primary role over 12 months	EMT N = 16,871 N (%)	AEMT N = 1,677 N (%)	Paramedic N = 14,787 N (%)	Total N (%)
Emergent response	12,185 (72.2)	1,385 (82.6)	11,516 (77.9)	25,086 (75.3)
Medical transport	1,466 (8.7)	90 (5.4)	790 (5.3)	2,346 (7.0)
Clinical services	1,467 (8.7)	96 (5.7)	864 (5.8)	2,427 (7.3)
Mobile integrated health/community paramedicine	141 (0.8)	7 (0.4)	144 (1.0)	292 (0.9)
Education and administration	424 (2.5)	43 (2.6)	986 (6.7)	1,453 (4.4)
Other	466 (2.8)	29 (1.7)	214 (1.4)	709 (2.1)
Missing	722	27	273	1,022

**Table 3:** Sub-category of roles respondents performed over the last 12 months AEMT: advanced emergency medical technicians; EMT: emergency medical technician; w/o: without

	EMT N = 16,871 N (%)	AEMT N = 1,677 N (%)	Paramedic N = 14,787 N (%)
Emergent response (immediate, with or without 9-1-1)			
Ground ambulance	6,635 (39.3)	984 (58.7)	8,657 (58.5)
Non-ambulance response (rescue squad, fire apparatus)	4,148 (24.6)	337 (20.1)	1,891 (12.8)
Aeromedical (fixed wing or rotor)	105 (0.6)	4 (0.2)	633 (4.3)
Law enforcement/security services/tactical	487 (2.9)	25 (1.5)	111 (0.8)
Clinical services (hospital/urgent care)			
Emergency department	758 (4.5)	42 (2.5)	451 (3.0)
Outpatient clinical space (physician’s office, clinic)	284 (1.7)	18 (1.1)	137 (0.9)
Hospital setting (hospital floor)	144 (0.9)	6 (0.4)	74 (0.5)
Intensive care unit (neonatal, pediatrics, adult)	27 (0.2)	5 (0.3)	39 (0.3)
Medical transport (non-emergent)			
Ground (interfacility transport, not critical care)	913 (5.4)	62 (3.7)	312 (2.1)
Ground (critical care transport)	182 (1.1)	13 (0.8)	220 (1.5)
Aeromedical transport	35 (0.2)	1 (0.1)	182 (1.2)
Ground (convalescent transport)	272 (1.6)	10 (0.6)	38 (0.3)
Mobile integrated health/community paramedicine			
Acute medical care	65 (0.4)	4 (0.2)	56 (0.4)
Prevention services	36 (0.2)	4 (0.2)	23 (0.2)
Coordination of services	3 (0.0)	0	18 (0.1)
Healthcare maintenance	14 (0.1)	1 (0.1)	17 (0.1)
None of the above			
Administrator (quality assurance, oversight)	189 (1.1)	18 (1.1)	532 (3.6)
Educator (for an agency or program)	235 (1.4)	25 (1.5)	454 (3.1)
Dispatcher/communications	92 (0.5)	7 (0.4)	41 (0.3)
Researcher	14 (0.1)	0	7 (0.0)
Missing	2,233	11	894

Our stratified analysis by sex noted further differences in demographics, work characteristics, and roles (Appendix A). Females were likelier to hold EMT certifications, whereas their male counterparts were often paramedics. Full-time employment was more common among males, who were 18.0% more likely to have such positions than females. However, females exhibited a greater inclination towards volunteer work. Regarding work environment roles, both predominantly worked in 9-1-1 service types, yet females were almost three times more likely than males to be in hospital/clinical services roles and almost twice as likely to work in medical transport (Table 4).

**Table 4:** EMS clinician work roles performed in the last 12 months stratified by sex

Primary role over 12 months	Female N = 8,728 N (%)	Male N = 24,498 N (%)
Emergent response (immediate, with or without 9-1-1)	5,711 (65.4)	19,297 (78.8)
Medical transport (non-emergent)	864 (9.9)	1,474 (6.0)
Clinical services (hospital, urgent care)	1,211 (13.9)	1,206 (4.9)
Mobile integrated health/community paramedicine	117 (1.3)	174 (0.7)
Education and administration	369 (4.2)	1,080 (4.4)
Other	274 (3.1)	434 (1.8)
Missing	182	833

## Discussion

Recognizing ongoing prehospital workforce shortages in the US, it’s crucial to clarify the diverse roles that EMS clinicians fulfill in emergency and non-emergency settings to fully grasp the available resources for emergent responses. In this evaluation, 24.7% of the sampled EMS workforce functions outside emergent response roles. At all certification levels, clinicians most commonly held emergent response primary roles and work in urban/suburban areas. While filling these roles, almost half of all respondents (48.8%) reported needing more than one job to make ends meet.

The original mission of EMS in the healthcare continuum was to provide emergent prehospital care [18]. However, in 1996, the need for changes in healthcare provision led to the EMS Agenda for the Future, which called for expanding the EMS role into public health and other healthcare fields [19]. In this study, we identified that 7.3% of EMS clinicians are not working in prehospital care but in hospital/clinical service roles. As EMS workforce shortages become primary concerns throughout the US, the relationship between these shortages and the expanded roles of EMS requires further evaluation [20,21]. Although this paradigm shift was identified as a need in the EMS Agenda for the Future in 1996, these findings may highlight possible unintended ramifications by reducing the proportion of available clinicians in emergent roles.

We also noted differences in work environment roles by sex. Previous work has noted disparities in the overall EMS workforce population with a higher representation of males as paramedics and EMTs [17]. In this work, we have also seen differences in roles by sex, with a higher propensity of females in clinical services and hospital settings and males working a higher proportion in fire-based EMS services. We also noted differences by sex in certification levels and education, which could impact career direction and trajectory. The reasons for these differences are unclear and may be multifactorial, including physical demands, perceived roles, and historical entry barriers [22,23]. Understanding these sex-based differences is crucial for EMS workforce planning since a diverse EMS workforce can enhance the overall quality of patient care by bringing varied experiences, skills, and perspectives to the field.

Limitations

Our study included civilian EMS clinicians recertifying with the National Registry. These numbers do not include EMS clinicians with only state certifications to practice and, therefore, may not fully represent the entire EMS population. Although there is no national sample of EMS clinicians, the National Registry database accounts for 54.9% of this population, with 489,495 clinicians of the estimated 891,322 EMTs, AEMTs, and paramedics from the 2020 National EMS Assessment [24,25]. Additionally, reporting bias is possible since we leverage self-reported data in this analysis. We had a response rate of 34.0% of the population, equating to almost 39,000 people. Though the response rate may appear low, the high number of respondents increases precision and helps mitigate bias [26,27]. In the larger framework of survey response, this is a higher response frequency than other national EMS samples, and response rates are similar to national estimates [28,29]. Additionally, per national guidelines, bias is evaluated by comparing non-responder populations (Appendix B) with external data [26,27]. Compared to the overall National Registry population, our sample is representative of the 2020 National Association of State EMS Officials EMS Workforce Estimate [25]. Our weighted analysis also showed that the surveyed population largely represented the overall national EMS workforce, with minor adjustments observed in age group distributions and racial composition (Appendix C). Differences between weighted and unweighted estimates were small, indicating that the sample reflected the broader national population. Finally, this evaluation examined the descriptive roles served by EMS clinicians and does not extrapolate on their impact or associations with workforce dynamics. Further assessments will need to explore the impact of EMS roles on workforce-reducing factors.

## Conclusions

This study underscores the complex landscape of EMS clinician roles within the US healthcare system, highlighting the significant portion of the workforce engaged in non-emergent and clinical services roles amidst ongoing prehospital workforce shortages. The necessity for EMS clinicians to hold multiple jobs to sustain themselves financially indicates systemic compensation and resource allocation issues that need urgent attention. These findings suggest the need for continued focus and attention on EMS roles, compensation structures, and population dynamics to ensure a resilient, diverse, and adequately supported EMS workforce.

## Appendices

Appendix A

**Table 5:** Characteristics by respondents’ sex AEMT: advanced emergency medical technician; EMT: emergency medical technician; GED: general education development; HS: high school; IQR: interquartile range

	Respondents’ Gender		
	Female n=8,728 (26.3) n (%)	Male n=24,498 (73.7) n (%)	Total n=33,226 n (%)
Age (median, IQR)	33 (26-43)	36 (29-46)	36 (28-46)
Certification level			
EMT	5,149 (59.0)	11,650 (47.6)	16,799 (50.6)
Advanced EMT	489 (5.6)	1,179 (4.8)	1,668 (5.0)
Paramedic	3,090 (35.4)	11,669 (47.6)	14,759 (44.4)
Race			
White, non-Hispanic	7,224 (82.8)	20,258 (82.7)	27,482 (82.7)
Other races	1,393 (16.0)	3,863 (15.8)	5,256 (15.8)
Missing	111	377	488
Education			
HS/GED or below	991 (11.4)	3,803 (15.5)	4,794 (14.4)
Some college	2,893 (33.1)	7,852 (32.1)	10,745 (32.3)
Associates	1,826 (20.9)	5,208 (21.3)	7,034 (21.2)
Bachelor’s or above	3,007 (34.5)	7,617 (31.1)	10,624 (32.9)
Missing	11	18	29
Experience years (median, IQR)	3 (2-4)	4 (2-6)	4 (2-6)
Full-time	5,344 (61.2)	19,523 (79.7)	24,867 (74.8)
Main EMS volunteer	1,635 (18.7)	2,458 (10.0)	4,093 (12.3)
Current EMS agency jobs			
1 job	6,510 (74.6)	17,627 (72.0)	24,137 (72.6)
2 or more jobs	2,218 (25.4)	6,871 (28.0)	9,089 (27.4)
Jobs to meet ends			
1 job	3,980 (45.6)	11,981 (48.9)	15,961 (48.0)
More than 1 job	4,541 (52.0)	11,674 (47.7)	16,215 (48.8)
Missing	207	843	1,050
Urbanicity			
Rural	1,282 (14.7)	2,210 (9.0)	3,492 (10.5)
Urban/Suburban	7,139 (81.8)	21,545 (87.9)	28,684 (86.3)
Missing	307	743	1,050

Appendix B 

**Table 6:** Population comparison between the study respondents, non-respondents, and overall National Registry population for emergency medical technician (EMT), advanced EMT, and paramedic certification levels HS: high school; GED: general education development; Govt: government; SD: standard deviation; * Asian, Black/African American, Hawaiian/Native Alaskan, Hispanic ethnicity

	Study Population (N = 33,335) n (%)	Surveyed Non-Respondents (N = 81,218) n (%)	2021 National Registry Population (N = 358,872) n (%)
Age (Mean, SD)	38 (11)	37 (11)	36 (11)
Sex			
Male	24,498 (73.5)	57,413 (70.7)	241,791 (67.4)
Female	8,728 (26.2)	23,433 (28.9)	104,124 (29.0)
Missing	109	372	12,957
Race			
White, non-Hispanic	27,522 (82.6)	63,201 (77.8)	262,645 (73.2)
Other*	5,262 (15.8)	16,050 (19.8)	74,888 (20.9)
Missing	551	1,967	21,339
Education			
	4,806 (14.4)	13,059 (16.1)	44,919 (12.5)
Some college	10,769 (32.3)	25,639 (31.6)	81,453 (22.7)
Associates	7,054 (21.2)	16,014 (19.7)	43,549 (12.1)
Bachelor’s or above	10,675 (32.0)	26,400 (32.5)	66,160 (18.4)
Missing	31	106	122,791
Agency type			
Fire	14,937 (44.8)	31,551 (38.8)	90,042 (25.1)
Private	7,188 (21.6)	12,110 (14.9)	42,189 (11.8)
Govt non-fire	3,979 (11.9)	7,669 (9.4)	29,480 (8.2)
Hospital	4,077 (12.2)	6,524 (8.0)	19,960 (5.6)
Missing	3,154	23,364	177,201

Appendix C 

**Table 7:** Comparison of weighted and unweighted proportions across various demographic and occupational characteristics for emergency medical technicians (EMTs), advanced EMTs (AEMTs), and paramedics HS: high school; GED: general education development; Govt: government

	EMT weighted 17.3%	EMT unweighted 17.5%	AEMT weighted 1.7%	AEMT unweighted 1.7%	Paramedic weighted 15.2%	Paramedic unweighted 15.3%	Total weighted 34.1%	Total unweighted 34.6%
Age groups								
18-27	27.0%	32.1%	21.6%	24.0%	9.5%	9.8%	19.8%	21.8%
28-37	32.1%	31.4%	36.7%	34.0%	36.4%	36.8%	34.0%	33.9%
38-47	22.4%	19.0%	23.3%	23.0%	28.3%	27.4%	24.8%	23.0%
48-57	12.9%	11.8%	13.4%	13.1%	19.6%	19.5%	15.6%	15.3%
58-67	4.7%	4.8%	4.2%	5.0%	5.7%	5.9%	5.0%	5.3%
68-77	0.8%	0.9%	0.8%	0.8%	0.5%	0.6%	0.7%	0.7%
78-85	0.0%	0.1%	0.0%	0.1%	0.0%	0.0%	0.0%	0.0%
Sex								
Female	26.3%	30.7%	26.7%	29.3%	21.1%	20.9%	24.3%	26.3%
Male	73.7%	69.4%	73.3%	70.7%	78.9%	79.1%	75.7%	73.7%
Race								
White, non-Hispanic	79.4%	80.2%	83.7%	87.0%	87.5%	87.9%	82.9%	84.0%
Everyone else	20.7%	19.8%	16.3%	13.0%	12.5%	12.1%	17.2%	16.1%
Number of EMS jobs								
1 job	80.1%	80.2%	71.5%	67.3%	70.9%	68.3%	75.7%	72.7%
2+ jobs	19.9%	19.8%	28.5%	32.7%	29.1%	31.7%	24.3%	27.3%
Education								
HS/GED or below	20.2%	18.2%	20.0%	17.0%	10.9%	9.8%	16.3%	14.4%
Some college	32.6%	34.2%	37.5%	37.8%	30.6%	29.6%	32.2%	32.3%
Associates	15.5%	15.0%	17.0%	17.4%	27.9%	28.7%	20.7%	21.2%
Bachelor’s or above	31.8%	32.6%	25.5%	27.9%	30.6%	31.9%	30.9%	32.1%
Agency type								
Fire	9.1%	11.4%	9.2%	10.4%	13.0%	13.6%	10.8%	12.3%
Hospital	52.0%	45.9%	55.2%	51.9%	45.3%	43.3%	49.0%	45.1%
Govt non-fire	11.1%	10.3%	10.9%	11.2%	13.5%	14.0%	12.1%	12.0%
Private	19.9%	23.4%	20.2%	21.3%	19.4%	19.8%	20.0%	21.7%
Other	7.9%	9.0%	4.5%	5.1%	8.8%	9.3%	8.1%	9.0%
